# Supplementary material for: RSV competes with the host for translational machinery without a host shutoff strategy
Source: bioRxiv. 2026 Sep 1:2026.08.31.748072. Preprint. [Version 1] doi: 10.64898/2026.08.31.748072 (PMC13684578; doi:10.64898/2026.08.31.748072)

bioRxiv preprint doi: https://doi.org/10.64898/2026.08.31.748072; this version posted September 1, 2026. The copyright holder for this preprint (which was not certified by peer review) is the author/funder. This article is a US Government work. It is not subject to copyright under 17 USC 105 and is also made available for use under a CC0 license.

Supplementary Figure Legends

Figure S1. Viral, IFN and ISG transcripts are upregulated during RSV infection.

(A) Immunofluorescence staining of mock- and RSV-infected cells after 24 h demonstrates a high infection rate. DAPI staining identifies nuclei (in blue), PABPC1 outlines the cytoplasm (in red) and polyclonal RSV antibody detects RSV infection (in green).

(B) Western blot comparing RSV protein levels at different timepoints. RSV proteins were detected with a polyclonal RSV antibody and GAPDH serves as a loading control.

(C) Quantification of the relative abundance of ERCC mRNAs, shown as a percentage of the total of raw read counts that came from host, virus and ERCC mRNAs. A paired t-test was performed to compare ERCC mRNA abundance between uninfected and infected conditions for each timepoint separately. Each datapoint indicates the value from a biological replicate with bars representing the mean and error bars indicating the standard deviation.

(D) Volcano plot showing upregulation of viral mRNAs during infection. RSV infection induces a strong IFN response, while VACV infection with WT virus does not. ISG response appears to decrease due to loss of basal level of expression. Adjusted p values (padj) and log2 fold changes were calculated by DESeq2 using the Benjamini–Hochberg procedure for ERCC-normalized raw read counts. Significance cutoff was set at padj < 0.05 and log2 fold change > 1 or < -1. IFN: interferon, ISG: IFN-stimulated genes. S: significant, NC: not significant.

Figure S2. Translation and rRNA levels remain unchanged following RSV infection.

(A) Western blot analysis validating the SUnSET assay by reducing translation with cycloheximide (CHX) and arsenite treatment. Cells were incubated with puromycin (10 µg/mL) for 10 minutes prior to harvest to label nascent proteins. CHX (100 µg/mL) was added 10 minutes before puromycin, as a negative control, while arsenite treatment started 50 minutes before puromycin addition. Nascent proteins were detected using an anti-puromycin antibody and β-actin served as a loading control. Detection of eIF2α and phosphorylated eIF2α-P confirmed arsenite-induced translation shutdown. Note that blot shows nascent proteins, so weights correspond to partially-completed protein.

(B) SUnSET assay comparing uninfected and RSV-infected (24h, MOI 3) samples by western blot analysis. Nascent proteins were detected using an anti-puromycin antibody and RSV infection was confirmed using a polyclonal RSV antibody. Nascent protein levels are similar between uninfected and infected cells.

bioRxiv preprint doi: https://doi.org/10.64898/2026.08.31.748072; this version posted September 1, 2026. The copyright holder for this preprint (which was not certified by peer review) is the author/funder. This article is a US Government work. It is not subject to copyright under 17 USC 105 and is also made available for use under a CC0 license.

(C) Quantitative analysis of rRNA levels by TapeStation analysis. Cells were infected with RSV (MOI 3), treated with 25 nM Torin1 or serum starved for 24 hours. Live cell counts were obtained from each well, and RNA was extracted after adding a fixed amount of 60-mer RNA spike-in per well. RNA levels for spike-in and rRNA were quantified and rRNA level per cell was computed and found to remain similar between conditions. Each datapoint indicates the value from a biological replicate with bars representing the mean and error bars indicating the standard deviation.

Figure S3. Quality control of timecourse ribo-seq and RNA-seq datasets.

(A) Ribosome footprint size analysis for uninfected and infected datasets. The probability density is plotted for main ORF, start and stop codons, which serves as a quality control check for the ribosome profiling data.

(B) PCA analysis for RNA-seq and ribo-seq timecourse datasets separates datasets by transcriptome and translatome (PC1) and infection duration (PC2).

Figure S4. Heatmap of transcriptome-wide mRNA distribution across sucrose gradient fractions. The distribution of host transcripts along the sucrose gradient is shown as a heatmap and transcripts are sorted by main ORF length, with the shortest transcript at the top. Under uninfected conditions, transcripts with shorter ORFs are found in lighter polysome fractions while longer transcripts are in heavier polysome fractions, as expected. During infection, fewer host transcripts are found in polysomes.

Figure S5. mRNA distribution of viral mRNAs and ribosome density of uninfected cells.

(A) Line graphs showing the distribution of individual viral mRNAs across the sucrose gradient. The black line represents the average, and standard deviation is shown as shaded areas. Host mRNAs with similar ORF lengths (±30 nt) are colored according to their most abundant location within the gradient (80S: light orange, Lpol: dark orange, Mpol: green, Hpol: blue). Pie charts show the proportion of dominant fraction of each transcript across the gradient. The ORF length of the viral transcripts is indicated above as well as the number (n) of host transcripts included in the analysis. 80S: monosome fraction, Lpol: light polysomes, Mpol: middle polysomes and Hpol: heavy polysomes.

(B) Ribosome density of individual host mRNAs in uninfected cells, calculated as number of ribosomes per 100 nt ORF length. Graph demonstrates that shorter ORFs tend to have higher ribosome density, as expected.

Figure S6. Depletion of eIF4E reduces global translation of host and viral genes.

(A) Schematic overview of regulation of cap-binding protein eIF4E by 4E-BP1.

bioRxiv preprint doi: https://doi.org/10.64898/2026.08.31.748072; this version posted September 1, 2026. The copyright holder for this preprint (which was not certified by peer review) is the author/funder. This article is a US Government work. It is not subject to copyright under 17 USC 105 and is also made available for use under a CC0 license.

(B) Western blot and quantification confirm eIF4E knockdown with GAPDH serving as a loading control. NT: non-targeting. Each datapoint indicates the value from a biological replicate with bars representing the mean and error bars indicating the standard deviation.

(C) Depletion of eIF4E shows only a slight reduction in 4E-BP1 protein (shown by western blot). β-actin served as a loading control. NT: non-targeting. Each datapoint indicates the value from a biological replicate with bars representing the mean and error bars indicating the standard deviation.

(D) Polysome profiling comparing non-targeting (NT) and eIF4E siRNA transfected cells shows reduction in the polysome to monosome ratio, indicating a global reduction in translation.

(E) Western blot analysis shows similar levels of RSV proteins when comparing non-targeting (NT) and eIF4E siRNA transfected cells. GAPDH shown as a loading control. Each datapoint indicates the value from a biological replicate with bars representing the mean and error bars indicating the standard deviation.

Figure S7. RSV translation decreases following Torin1 treatment.

(A) Schematic overview of 4E-BP1 dephosphorylation which occurs following Torin1 treatment. Dephosphorylated 4E-BP1 has a high affinity for cap-binding protein eIF4E (shown on left), resulting in decreased translation initiation.

(B,C) Western blot analysis of 4E-BP1 phosphorylation status following Torin1 treatment and RSV infection (B). Blot shows results obtained with three antibodies (bottom two blots are phospho-specific). Subtle shifts in band position correspond to phosphorylation status. As expected, Torin1 treatment resulted in complete dephosphorylation of 4E-BP1 (no bands in bottom two blots in lane 2 and 4). RSV infection resulted in a slight reduction in phosphorylated 4E-BP1 on both phosphorylation sites (lane 3 versus 1 of blots, see reduction in bottom two blots), as quantified in (C). Each datapoint indicates the value from a biological replicate with bars representing the mean and error bars indicating the standard deviation.

(D) Differential expression analysis as computed by DESeq for all mRNAs shows the effect of Torin1 treatment in infected cells, for RNA-seq and ribo-seq datasets. The RNA abundance of 5′-TOP and RSV mRNAs was slightly increased, while the ribosome association was strongly reduced for 5′-TOP mRNAs.

(E) Volcano plot showing statistical significance for changes in TE of mRNAs after Torin1 treatment of infected cells (computed by DESeq2). Data show that 5′-TOP and RSV mRNAs decrease in TE following Torin1 treatment. Adjusted p values (padj) and log2 fold changes were calculated by DESeq2 using the Benjamini–Hochberg

bioRxiv preprint doi: https://doi.org/10.64898/2026.08.31.748072; this version posted September 1, 2026. The copyright holder for this preprint (which was not certified by peer review) is the author/funder. This article is a US Government work. It is not subject to copyright under 17 USC 105 and is also made available for use under a CC0 license.

procedure for ERCC-normalized raw read counts. Significance cutoff was set at padj < 0.05 and log2FC > 1 or < -1. S: significant, NC: not significant.

Figure S8. Validation of LARP1 IP and KO by comparisons between non-TOP, 5′-TOP and viral mRNAs.

(A) Western blot analysis to confirm immunoprecipitation of LARP1 protein. Immunoprecipitation was performed with a LARP1 specific antibody and an IgG negative control. LARP1 was detected in input, flow-through (FT) and LARP1-IP elution samples. LARP1 protein was not detected following IgG-IP elution. GAPDH was used as a loading control and negative control (no signal in IP elution lanes). The secondary antibody used to detect primary antibodies also detected the heavy chains of the antibodies used in the IPs and are annotated on the blot (IgG heavy chain in all elution lanes).

(B) Western blot confirming LARP1 KO cell lines. Total protein was detected using Ponceau staining.

(C) Differential expression for all individual mRNA in WT and *LARP1* KO cells to determine the effect of Torin1 treatment in infected cells, for RNA-seq and ribo-seq datasets. The reduction in ribosome association of 5′-TOP mRNAs did not occur in absence of LARP1.

(D) Motif logo of the first 15 nucleotides of an mRNA for all transcripts, 5′-TOP mRNAs and RSV mRNAs. As expected, a strong 5′ terminal oligopyrimidine tract (TOP) was observed in 5′-TOP mRNAs. This motif was absent in viral mRNAs.

Figure S9. GADD34 requires phosphatase activity for eIF2α-P dephosphorylation during RSV infection.

(A) Schematic representation of plasmid DNA encoding GADD34 overexpression mutants. Deletion of the PP1 domain (ΔPP1) results in deficient binding to phosphatase PP1. Deletion of the PEST1 domain (ΔPEST1) results in decreased binding to eIF2α-P. Both deletions are expected to decrease the ability of GADD34 to decrease eIF2α-P dephosphorylation.

(B) Western blot analysis of rescue experiments in GADD34 KO cells. eIF2α phosphorylation levels are increased in GADD34 KO cells following RSV infection. Overexpression of GADD34 reduced eIF2α phosphorylation levels back to similar levels as WT cells. In contrast, overexpression of ΔPP1 and ΔPEST1 GADD34 mutants did not reduce eIF2α phosphorylation to background levels. β-actin and total eIF2α served as loading controls and GADD34 as KO control and plasmid overexpression control.

bioRxiv preprint doi: https://doi.org/10.64898/2026.08.31.748072; this version posted September 1, 2026. The copyright holder for this preprint (which was not certified by peer review) is the author/funder. This article is a US Government work. It is not subject to copyright under 17 USC 105 and is also made available for use under a CC0 license.

Supplementary Figure S1

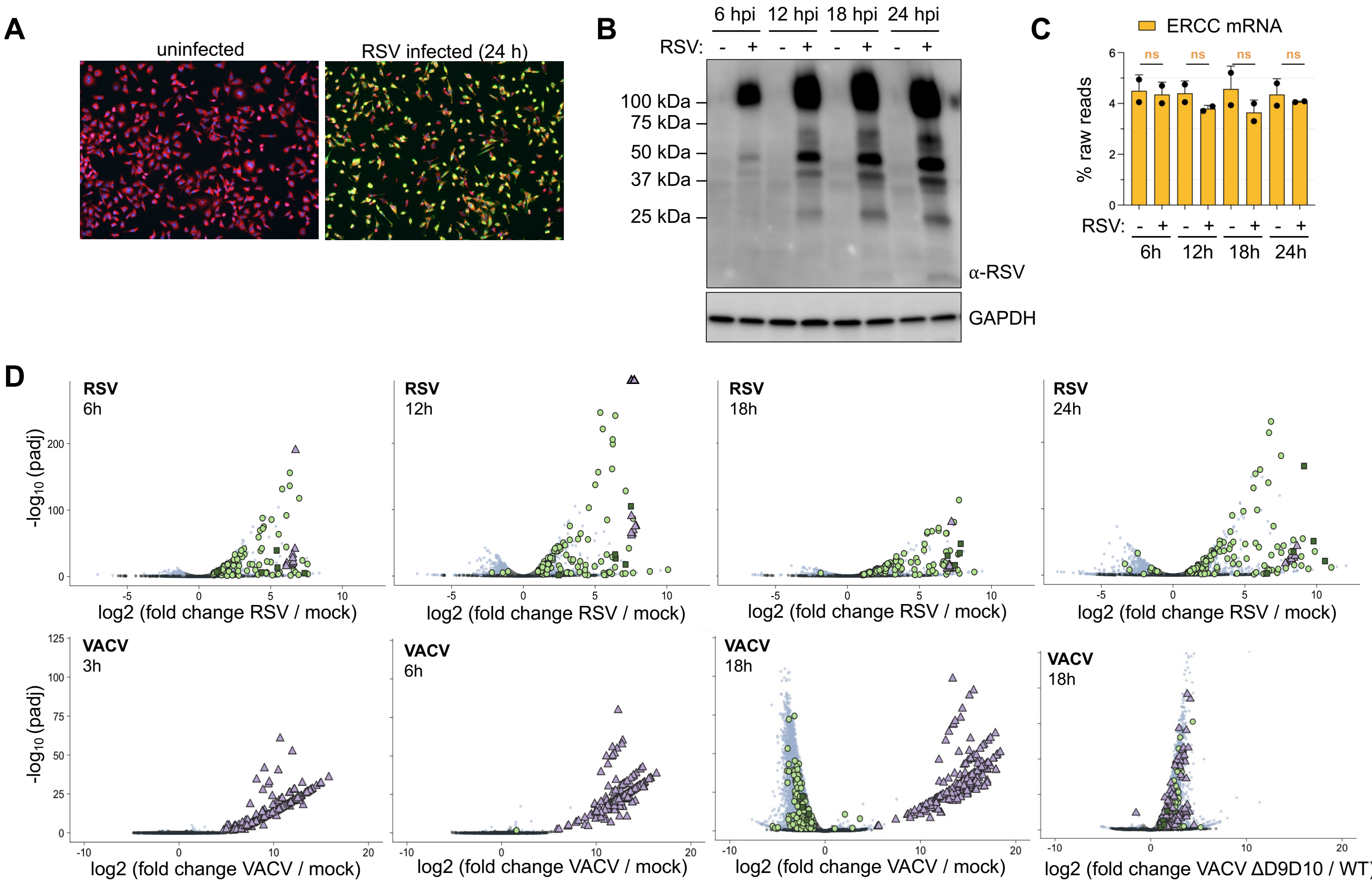

bioRxiv preprint doi: https://doi.org/10.64898/2026.08.31.748072; this version posted September 1, 2026. The copyright holder for this preprint (which was not certified by peer review) is the author/funder. This article is a US Government work. It is not subject to copyright under 17 USC 105 and is also made available for use under a CC0 license.

Supplementary Figure S2

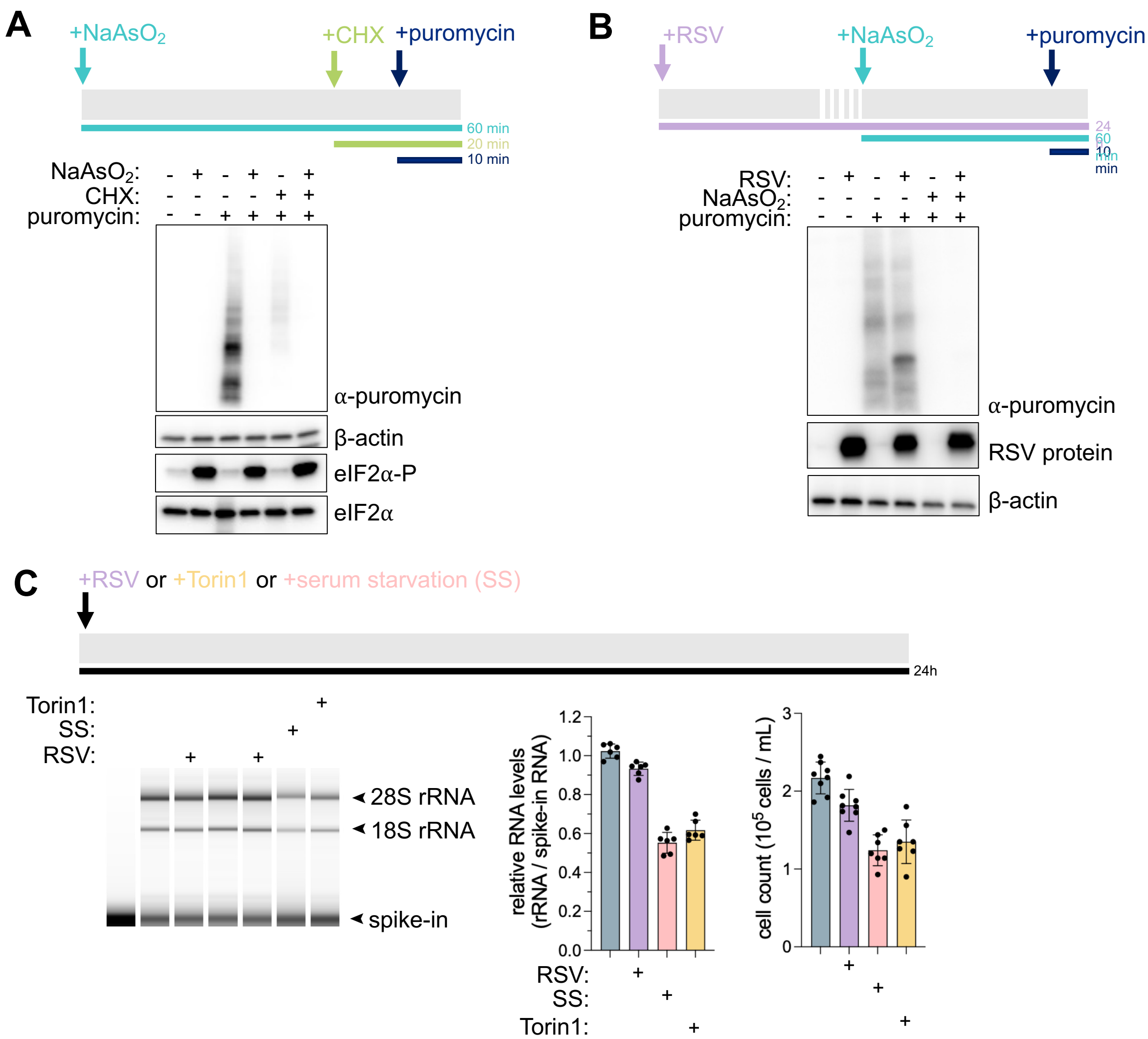

bioRxiv preprint doi: https://doi.org/10.64898/2026.08.31.748072; this version posted September 1, 2026. The copyright holder for this preprint (which was not certified by peer review) is the author/funder. This article is a US Government work. It is not subject to copyright under 17 USC 105 and is also made available for use under a CC0 license.

Supplementary Figure S3

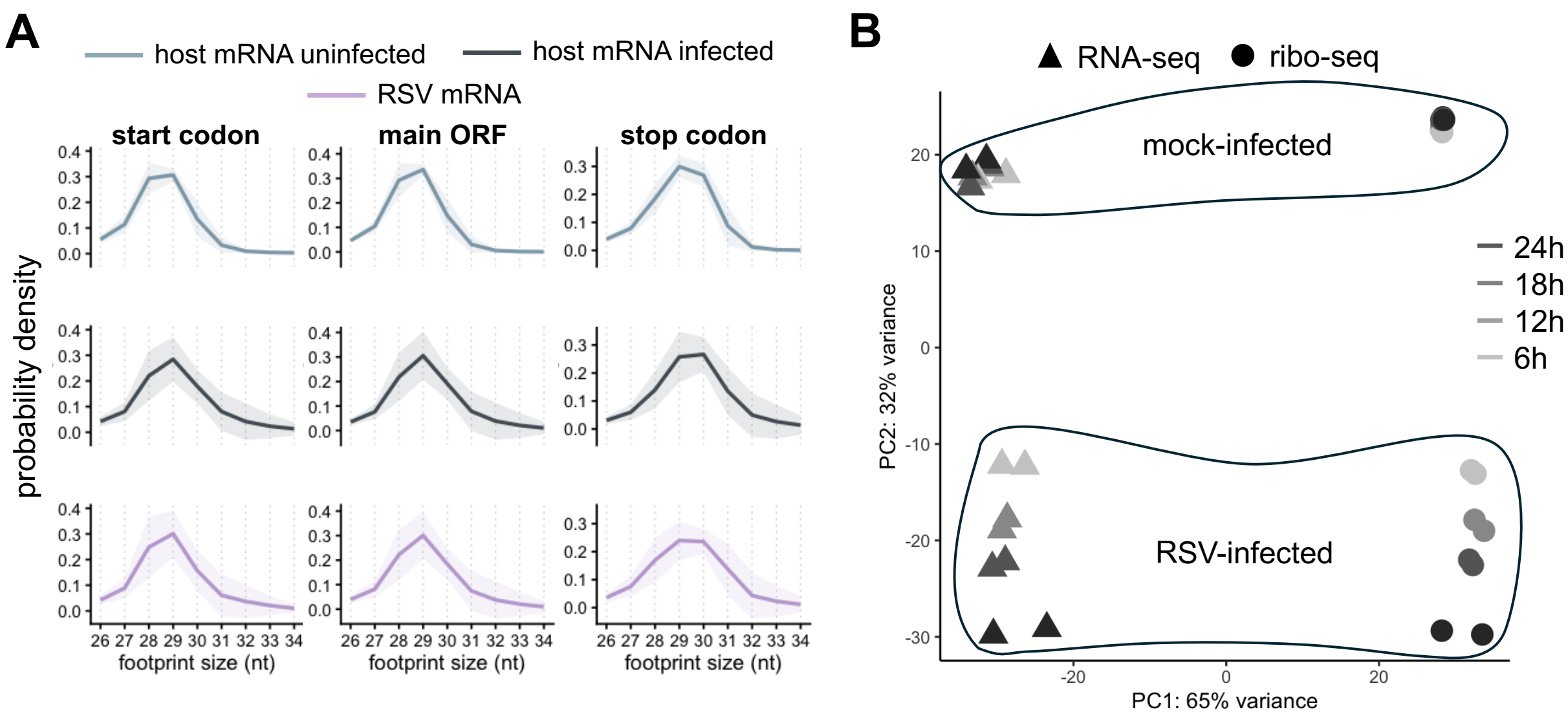

Supplementary Figure S4

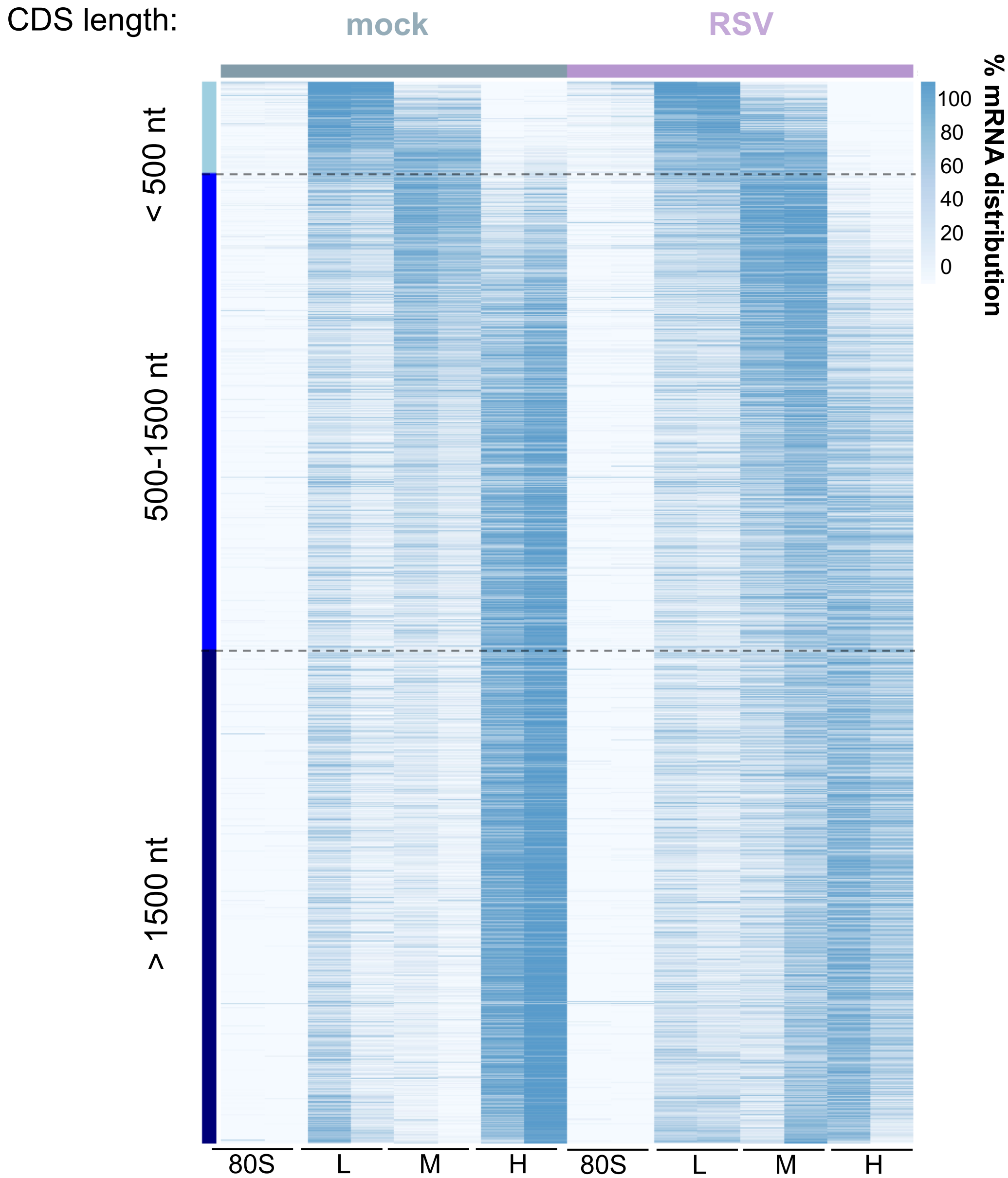

Supplementary Figure S5

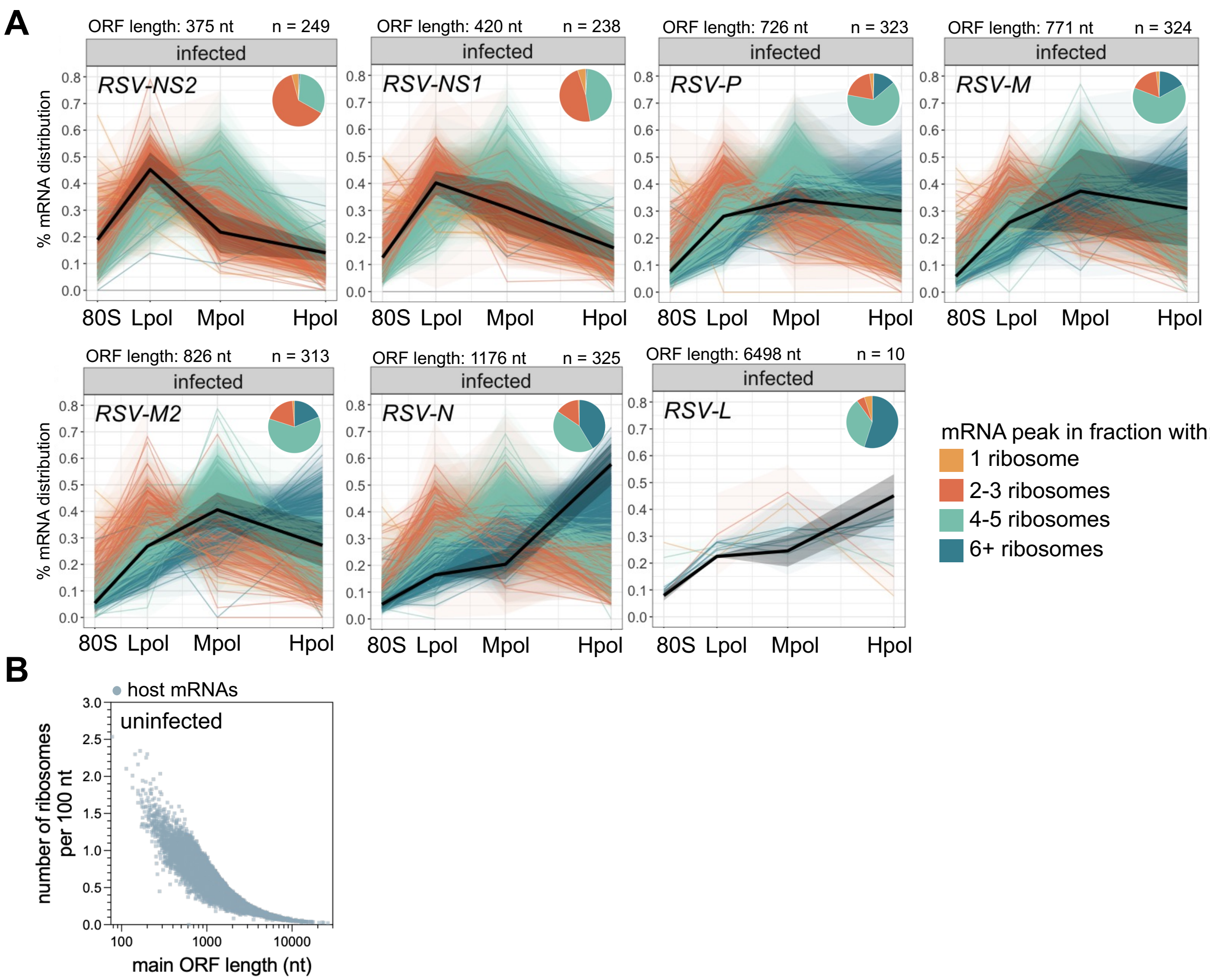

Supplementary Figure S6

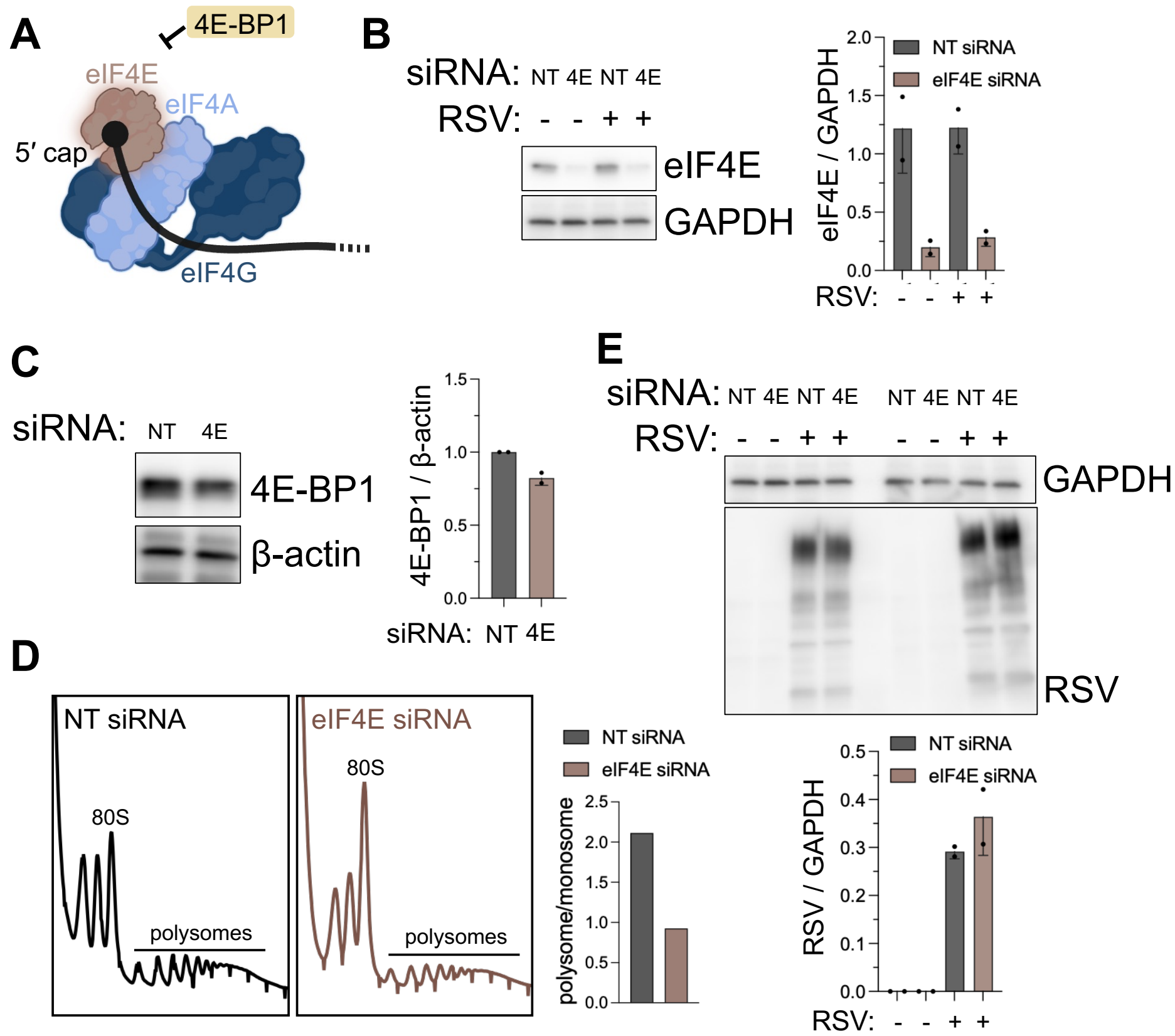

Supplementary Figure S7

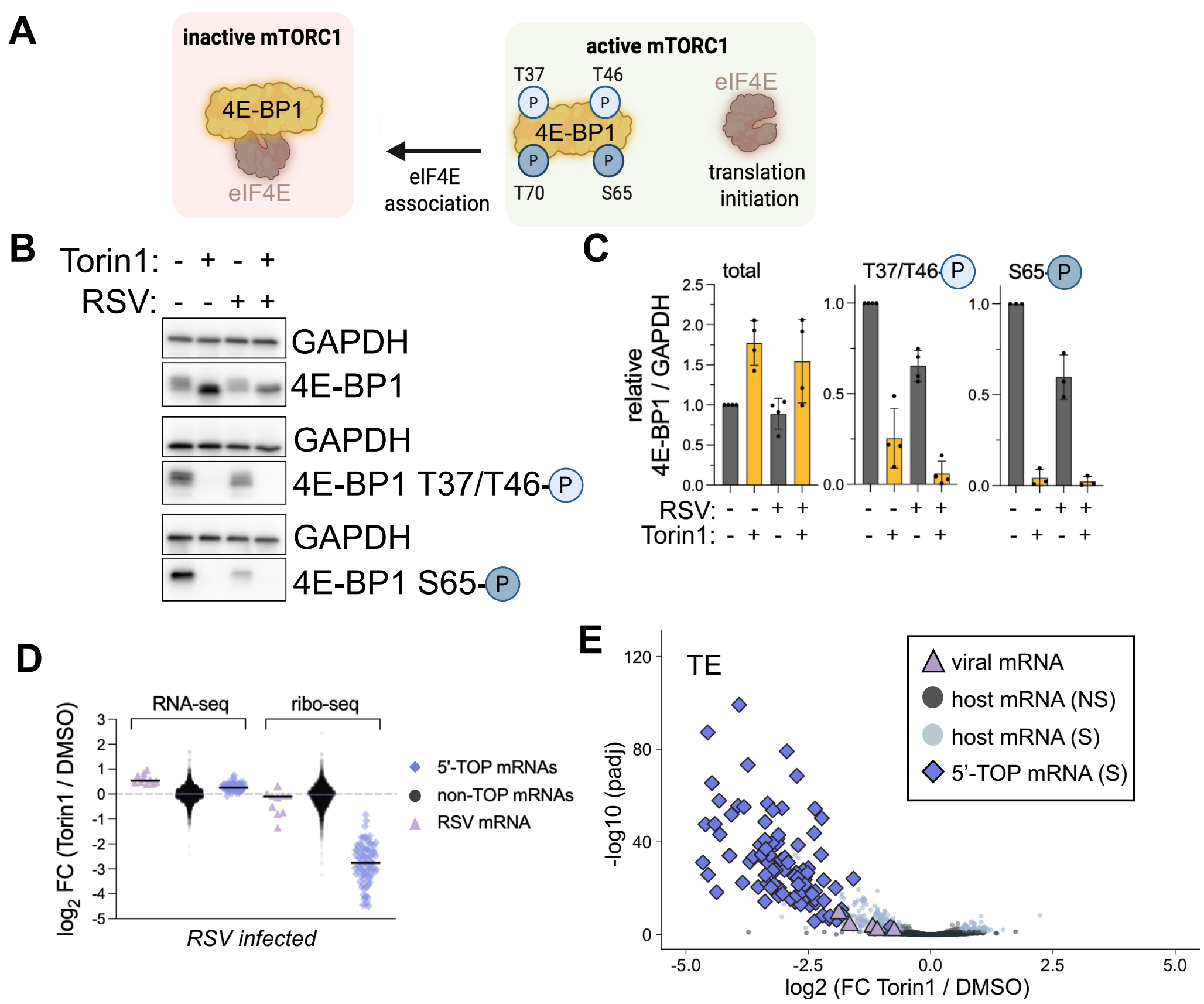

Supplementary Figure S8

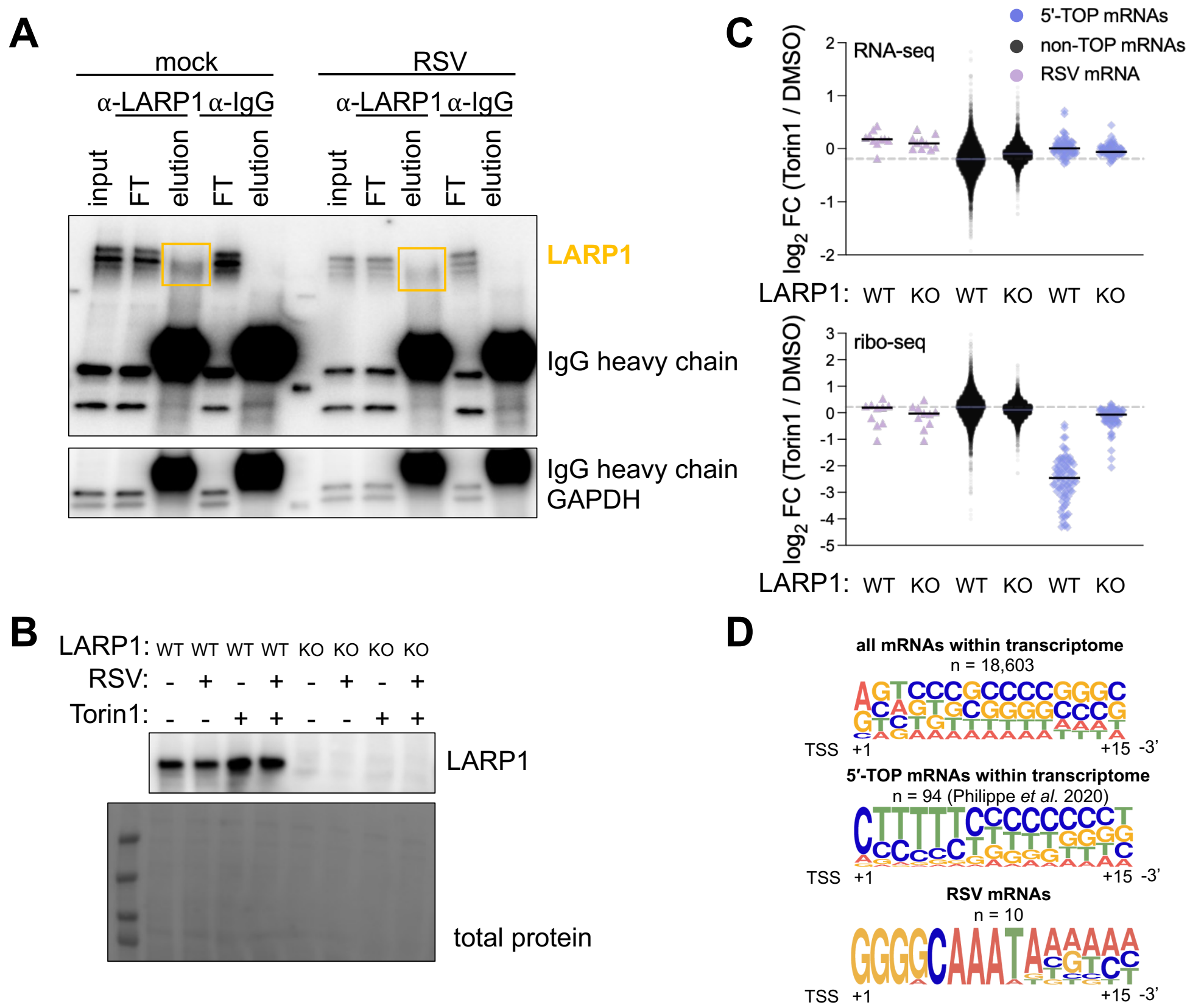

Supplementary Figure S9

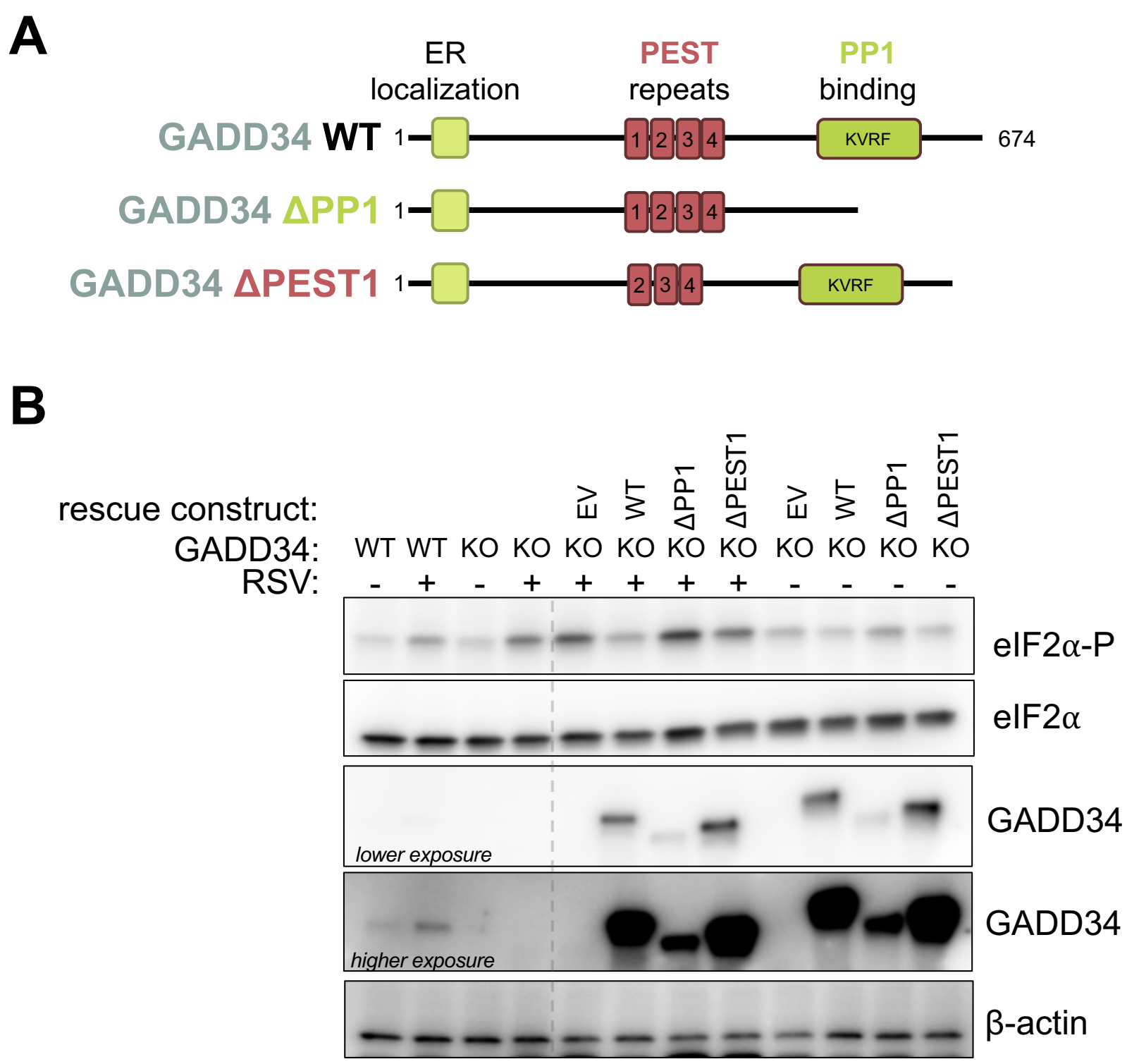

Supplement: Supplementary file 1 [file NIHPP2026.08.31.748072v1-supplement-14.pdf]
